# Palliative care making a difference in rural Uganda, Kenya and Malawi: three rapid evaluation field studies

**DOI:** 10.1186/1472-684X-10-8

**Published:** 2011-05-12

**Authors:** Liz Grant, Judith Brown, Mhoira Leng, Nadia Bettega, Scott A Murray

**Affiliations:** 1 Primary Palliative Care Research Group, Centre for Population Health Sciences: General Practice Section, The University of Edinburgh, Medical School, Teviot Place, Edinburgh EH8 9AG, UK; 2 Eastern Virginia Medical School, 431 New Hampshire Avenue, Norfolk, Virginia 23508, USA; 3 Cairdeas International Palliative Care Trust and Head of Palliative Care, Mulago Hospital and Makerere University, c/o Hospice Africa Uganda, PO BOX 7757, Kampala, Uganda; 4 Nadia Bettega, Photographer and anthropologist, 44c Sussex Way, London, N7 6RS, UK

## Abstract

**Background:**

Many people live and die in pain in Africa. We set out to describe patient, family and local community perspectives on the impact of three community based palliative care interventions in sub-Saharan Africa.

**Methods:**

Three palliative care programmes in Uganda, Kenya and Malawi were studied using rapid evaluation field techniques in each country, triangulating data from three sources: ***interviews ***with key informants, ***observations ***of clinical encounters and the local health and social care context, and routine data from local ***reports and statistics***.

**Results:**

We interviewed 33 patients with advanced illness, 27 family carers, 36 staff, 25 volunteers, and 29 community leaders and observed clinical care of 12 patients. In each site, oral morphine was being used effectively. Patients valued being treated with dignity and respect. Being supported at home reduced physical, emotional and financial burden of travel to, and care at health facilities. Practical support and instruction in feeding and bathing patients facilitated good deaths at home.

In each country mobile phones enabled rapid access to clinical and social support networks. Staff and volunteers generally reported that caring for the dying in the face of poverty was stressful, but also rewarding, with resilience fostered by having effective analgesia, and community support networks.

**Conclusions:**

Programmes were reported to be successful because they integrated symptom control with practical and emotional care, education, and spiritual care. Holistic palliative care can be delivered effectively in the face of poverty, but a public health approach is needed to ensure equitable provision.

## Background

Living and dying with incurable illness in poverty and pain is all too common in sub-Saharan Africa [1,2]. With minimal resources huge shortages of health workers, national health systems in a number of African countries have focussed primarily on preventive, curative and maternal health services, responding to a set of immediate (and development agency identified "best buys" in healthcare through their Essential (or Basic) health packages. In many countries minimal or no resources have been dedicated to supportive or palliative care. However various national and pan-African initiatives, such as Hospice Africa Uganda and the African Palliative Care Association are promoting palliative care in Africa [3], asserting that pain control is a human right [4]. The severity of the HIV and AIDS pandemic has triggered donor interest in palliative care in Africa, an interest which has never been greater, as evidenced by multiple small projects [5,6]. The World Health Assembly report of 2005 resolved, "Palliative care is an urgent humanitarian responsibility"[7]. Various palliative care programmes have developed, and national palliative care associations have emerged, but despite this positive change, palliative care is still only available to less than 5% of those in Africa who need it. Evaluation of the emerging initiatives and projects is vital to understand how people currently dying in poverty and pain might better live and die.

Our aim in this in-depth service evaluation was to describe patient, family and local community perspectives on three community based palliative care projects each in a country with high HIV prevalence. Two projects were in rural areas and one in a peri-urban setting. All three were community-based, involving volunteers and paid staff. Additional information on the sites visited can be found at: http://www.theworkcontinues.org/document.asp?id=1525.

The **Malawi **Home-Based Care Charitable Trust programme was established as an independent voluntary health organisation focussing on palliative care, following a needs assessment which identified that while there were a small range of social supports in the area, there were no clinical home based interventions for patients in need of palliative care. The programme employs two nurse directors, four nurses, six home based care assistants, a data manager and cleaner. Though separate from the government health service, the programme operates out of two government health centres in the Bangwe and Limbe peri-urban districts of Blantyre, the largest city in Malawi. Programme staff see their distinctive role as providing a clinical service in homes, with a focus on the patients' physical health.

In **Uganda**, the Kitovu Mobile palliative care service is based in Masaka, 120 km from the capital Kampala. The service covers four districts with a population of 1.5 million and is part of a larger faith based community programme of HIV support which focuses on home-based care, orphans and family support. This region was one of the areas hardest hit by the AIDS pandemic, especially the fishing communities on the shores of Lake Victoria. Poverty is endemic. Antiretroviral treatment availability has meant that now many people are living with, as well as dying from HIV.

In **Kenya**, the Maua palliative care programme was based in, and integrated with, the Community Health Department of Maua Hospital, a large well established rural Methodist church hospital in Igembe District, Eastern Province, about 240 km from Nairobi. Maua hospital serves approximately 700,000 residents. Since 2004, the palliative programme has treated people with HIV/AIDS (local prevalence is estimated at 12-15%) http://kehpca.org/wp-content/uploads/cover2.jpg and those with advanced cancers. Maua residents are generally poor, except for those involved in the *miraa (khat) *trade. *Miraa *brings to the community the additional challenges of a drug culture, including indiscriminate violence, especially to women and children, and wealth for a few amidst general poverty.

## Methods

At each site, three members of our multidisciplinary team which comprised of a medical anthropologist, a palliative care consultant who works in Uganda, a palliative care researcher and trainer, and a primary palliative care consultant accompanied by project staff and an independent translator, spent a week using rapid evaluation field techniques, supported by photographic ethnography to gain a range of information [8-10]. We used rapid evaluation methodology (REM), which was developed by the World Health Organisation (WHO) to assess the performance and quality of health services, identify operational problems, and assist in taking action. This method involves the collection of data using a range of skills and processes including observation of practice, interviews, and reviews of documents from different sources. We adopted three data collection activities, (outlined below) which provided the opportunity for verification through triangulation [11].

**1. Review of routine local information relevant to palliative care**. Team members read and summarised both published and unpublished documents relating to the three projects and the countries under study before field work began [3,12,13].

**2. Individual and small group interviews with patients, family members, staff and community leaders**. Potential interviewees were identified by the local programme team. A diverse sample were selected by the evaluators to explore the range of perspectives and needs, and to seek positive and negative perceptions. Interviews focused on how the programme impacted or not on patients' needs and wishes, and how it could be improved. Interviews were conducted in patients' homes or community settings. Using topic guides, one researcher led each discussion while another recorded notes manually, making a special effort to capture the colourful vernacular expressions often used by the respondents. Nearly all interviews in homes and communities were conducted in local languages with help from interpreters (who were, to avoid bias, not part of the project staff, but were hired and trained by the research team).

**3. Direct observations of palliative service provision and the general environment**. We observed and assessed the extent and quality of palliative care and counselling provided using a quality framework based on good practice which included the following domains: patient focussed, empathetic, provision of an holistic assessment (clinical, social, emotional, spiritual) appropriate listening skills and effective sensitive communication, confidentiality, and information giving. Observations were supported by photographs of the physical and socio-economic environment of care. They provided much graphic and detailed information, supporting and contextualising each person's unique narrative. At each interview site written permission was obtained for taking photographs from patients, their carers and staff, and for using photographs in future publications.

The field team collected data during a week at each site, between March and July 2009. Field notes were completed on site and initial data descriptions and analysis undertaken. Once all three field visits were completed, the data sets were analysed together using standard qualitative interview analytical techniques, interrogating the data for thematic correlates, and dissonances, and capturing linguistic expressions to convey individual, and communal beliefs, attitudes, and feelings [14]. All team members contributed to the analysis. Emerging themes were discussed in-country and were communicated by e-mail to other team members. Permission was granted to carry out these detailed evaluations from the relevant hospital board, and the Senior Management of the Charities responsible for delivering the palliative care programmes.

## Results

Table 1 details the number of patients, family carers, staff, volunteers, and community leaders interviewed, and the clinical care encounters we observed. Though the three programmes delivered palliative care through different models, and the contexts of delivery were different, patient and carer needs and their experiences of receiving palliative care were strikingly similar, as were the challenges that the three programmes faced. Palliative care in each programme was characterised by being more than just pain relief, or social care. Patients, carers and the local communities spoke of how the personalised package of care that they received made a difference in how they experienced and viewed death and dying, although further financial and practical support would have been greatly appreciated by many. A Ugandan patient captured the breadth and complexity of this multi-dimensional integrated care when he said, "*The palliative care people really helped me by bringing morphine and talking to me, bringing food and blankets for the children, and paying for me to go for the chemotherapy." (Ug M, HIV and KS) *The findings from each country are now presented in turn.

**Table 1:** People interviewed in the three case studies

	UGANDA Ug M = male Ug F = female FG = Focus group	KENYA Ken M = male Ken F = female FG = Focus group	MALAWI Mal M = male Mal F = female FG = Focus group	
**Patients**	8 patients at home	3 patients in hospital 6 patients at home (observed 4 clinical encounters)	7 patients at home (observed 4 clinical encounters) 9 patients in 2 focus groups HIV/AIDS, TB, breast cancer, KS	**33**

**Current home caregivers**	7	5 (son, son, husband & daughter, sister-in-law)	5 (daughter, wife, mother, sister, wife)	**17**

**Bereaved caregivers**	0	7 seen at home (wife, wife, daughter, wife, wife, niece & son) 2 at hospital (son & daughter)	1 (mother) daughter died of AIDS	**10**

**Volunteer (community) caregivers**	3 vol. in individual interviews	4 vol. in individual interviews 8 vol. in a focus group	10 volunteers in 2 focus groups (average length of stay of volunteers 4 years)	**25**

**Staff members of palliative care project**	6 individual interviews 10 staff in focus group	1 project in-charge 2 clinical officers 4 nurses	2 project in-charges, 4 nurses, 5 HBC Assistants, 2 support staff	**36**

**Project overseers**	0	7 members of hospital administration	1 project director, College of Medicine	**8**

**Community leaders**	3 (district councillor, district medical, officer, community leader)	17 in two focus groups	1 head of related community organisation	**21**

**TOTALS**	**37 people**	**66 people**	**47 people**	**150**

### Malawi

Most patients enrolled in the programme in Malawi were HIV+ and were treated at home. Many suffered from opportunistic infections and complications, such as Kaposi sarcoma or peripheral neuropathy. All patients were poor, with limited resources to purchase essentials such as food, warm clothes, gloves or sheets. Additional expenses such as transport to hospital were often met at the cost of family members going hungry, or school fees not being paid, some simply could not attend, *"Without home based care some people would certainly die as they would not have the strength to get to the hospital or health centre". (Mal Nurse)*

The lives of many patients, before the palliative care team intervention, were dominated by pain, severe sickness, and hopelessness. As one patient explained, *"Someone submitted my name to the volunteer and the volunteer came with a nurse to see me and she saw I was very sick. I said 'Better that I die', but the volunteer said, 'No, we have seen others like you and they get better.' I felt happy because up to then I had no way of help." (Mal M, HIV)*

The team brought knowledge about illnesses and encouraged access to services, changing community attitudes towards the dying. We heard many accounts of patients seeking treatments from traditional healers, often with costly consequences. When patients and their families finally stopped searching for cures, often when all their money had run out, hope disappeared and with it any ongoing treatment to alleviate sickness, or reduce pain. There was no alternative but to wait for death.

One patient with AIDS who lived on the outskirts of Blantyre told us, "*My relatives wanted me to go back to my home village to die, but there are no injections available in the village and I would have died if I had gone there. Now I am cared for right here." (Mal M, HIV)*

Practical help was given to both patients and carers, "*Towards the end of her life"*, said one mother whose daughter had died of AIDS, *"my daughter was unable to go to the toilet or feed herself. Even her eyes were jaundiced. The home based care people all came together to help. They even provided me with gloves so that I could use them to lift the soiled linen. They talked to me about my daughter." (Mal FC HIV pt - now deceased) *Others explained how the team provided guidance in how to care, reassurance that the family were giving good care, and support to keep on caring. *"I know the team will visit me*, said a wife, *"I will be very happy because they will counsel me on how to take care of him". (Mal FC HIV pt) *Patients and carers contrasted the care they received from the palliative care team with that they received in hospital, "*The home based care team spend a lot of time with me at home, but the hospital people are always in a hurry*". (Mal F, HIV) Another patient noted, *"When the volunteers come into my home, they treat me with ulemu (due respect, dignity)." *(Mal M (FG) HIV) Clinical and emotional needs were intricately connected to the need for food, basic shelter, warmth, and school fees, which frequently dominated the thoughts of patients and carers. Some patients were critical of the failure of teams to provide comprehensive care, *"My house leaks when it rains, and the bedding is not enough. I am very cold in the evenings, especially this month. I wish the team could help mend my house" said *Zora, a patient in Malawi. (Mal F, HIV + TB)

Volunteers were the backbone of the care team, and part of their effectiveness was their closeness to the community they worked in, "*The different chiefs held meetings and introduced the health volunteers. So we knew them as people, as neighbours. Then later, when we got sick, we already knew about the volunteers and we called for them." (Mal M, (FG) *They became linchpins in an informal referral system, "*The volunteers are the most important people, because they are the first ones to see us. Volunteers hear that someone is sick, and they come tapping at the door"*. (Mal F, (FG) HIV)

Volunteers referred patients to the palliative care nurses and their paid assistants, the home based care assistants for more specialised care, and they in turn referred through to hospital care. As one nurse explained, *"We are the bridge between the hospital and the community". (Mal Nurse)*

Providing care of such intensity and constancy was demanding, and endless. The joy of seeing patients feel better was countered by a sense of frustration at not being able to fully manage patients needs, both because of limited resources but also because of the lack of integration of the different health care streams. *"Yes"*, said one nurse, *"there are two systems at work"*. (Mal staff leader) She explained how the team, in continuous contact with the patients knew first hand their problems of peripheral neuropathy, while the HIV clinic, with limited knowledge of the patient, wanted tests to prove the diagnosis. Patients suffered from the double, sometimes triple systems. As one patient, receiving palliative care from the team explained, "*I get my AIDS medicines from the health centre but I have to go to hospital for my drugs for Kaposi's sarcoma from the hospital". (Mal M, HIV +KS)*

Volunteers supported their patients and families throughout the duration of the illness, and this was enhanced by the innovative way mobile phones were used to deliver palliative care. As one volunteer explained, *"Nurses really help us volunteers when we call them. We flash them from our cell phones, and they ring us back, and if we need them they come" ( Mal Vol) *Flashing - calling the number but not letting the call go through - did not cost anything, but alerted the nurse to the need for a conversation to give advice and assistance thus creating an ongoing communication line.

The availability of treatment for patients with AIDS meant that the palliative care programme, while still caring for terminally ill patients, also found itself addressing a changing trajectory and changing life expectancy. New issues were emerging such as the need for jobs and rehabilitation. Some patients were frustrated feeling that it was livelihood help rather than just clinical or home care support that was really needed" *If *"*If I could be assisted to do a small scale business, that would help me." (Mal F, HIV)*

### Uganda

The Kitovu palliative care programme functions through a freestanding mobile clinic system, part of an extensive and well established network built up by a church based organisation. The programme sees many people with AIDS and cancers at home.

**Figure 1:**
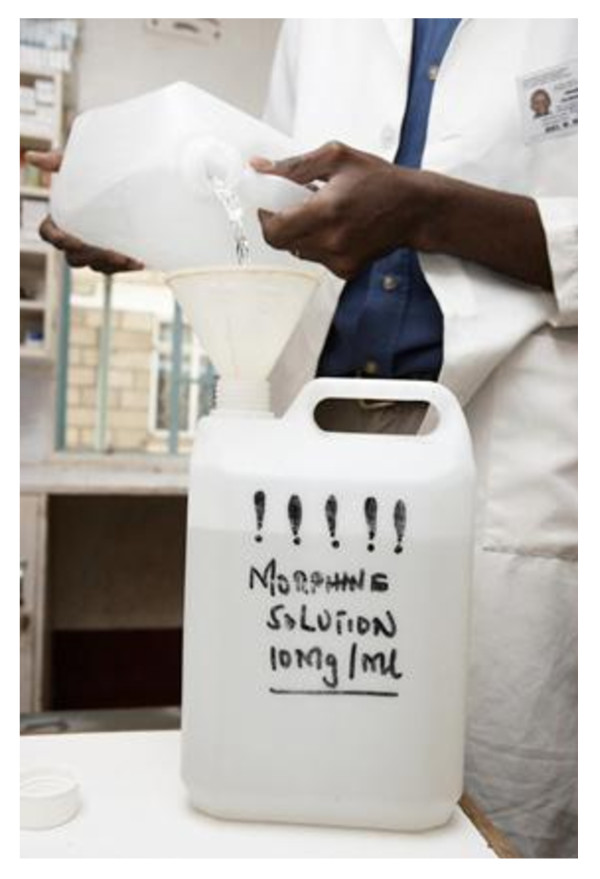
**Oral morphine solution being made from powder in the hospital**.

Pain dominated the lives of many patients before the palliative care team started. One patient speaking of the difference explained, "*The morphine brought back my happiness. I have no words to express my gratitude"*. (Ug F, Cancer) Her mother added, *"Lillian couldn't sleep day or night and could not be left alone, until God brought these people to see her." (Ug Carer of daughter HIV) *Rose a staff nurse noted, *"Now we are observing that something can happen to let people die in peace... People died in agony before, but now we realise the importance of controlling pain". (Ug Staff) *A Ugandan volunteer also noted the difference in how people died, *"Now (with morphine) patients die with dignity." *(Ug Vol). Figure 1 illustrates oral morphine solution being prepared from powder. 

But providing pain relief was just one part, albeit a vital part, of this programme which involved emotional, spiritual, social and practical care. A Ugandan nurse told us of a patient whose wound smelt so badly that no one would go near. The patient said, "*Nurse, you don't want to touch it"*. "*But we do touch and treat their wounds....This touching helps put a smile on people's faces"*. (Ug Nurse) Other staff noted of their presence, *"We instil a sense of hope....Before, hope was lost in pain." (Ug Nurse)*

The relationship that team members built with patients was important in tackling difficult and often hidden issues. "*One woman has cancer of the cervix, and sex is very painful for her, but her husband will not desist. We have to advise her to take oral morphine prior to sex. It hurts us when we see this, but we have to try and help the woman and talk to the husband". (Ug Nurse)*

Poverty shaped how people died. As in Malawi, also in Uganda, already poor patients were made poorer by illness, and by futile attempts to gain cure. One woman caring for her nephew said, *"I took him to over 20 traditional healers, .... I spent over a million shillings (£335) on medical care that failed to heal him. To pay for that, I took my own children out of school and sold their inheritance. I sold our land, cows, goats, chickens*." (Ug Carer for HIV nephew) This meant, as a team member explained, "*By the time people get to our programme, they have used up all their money on transport, medical treatment and witch doctors. "After we care for them, the people say, 'You are a god, you have saved us'"*. (Ug Staff) The multi-disciplinary, multi-tasking nature of the Kitovu palliative care team contributed to the delivery of care responsive to patients and carers' needs. Volunteers, again the backbone of the service, were also trained to give advice on family planning, health promotion, making wills, and using herbal medicines: this allowed them to be well integrated in and useful to the community. Volunteers were often recruited through churches, and they called on their church networks for support. Described by some community leaders as "community consultants", the volunteers were often active at the heart of the communities. A local politician in Uganda said, *"The community now identify patients and alert the volunteers, who then alert Kitovu Mobile. They give painkillers, financial support, help keep children in school, give medical things such as gloves and plastic sheeting"*.

Staff and patients spoke of how the presence of the palliative care team was changing community attitudes to death and dying. Though the task was uphill, as one nurse explained, "*We try to teach the reality, but people expect cure and treatment. They expect miracles*", the team though spoke of the trust that patients and carers had in them, and the acceptance that care can be much more than cure. The palliative care team brought expert knowledge about the nature and the effects of diseases into local communities, where such knowledge was previously absent, and while stigmatisation still occurred, especially for patients cancers, there was at least a greater acceptance.

The palliative care nurses, aides and volunteers had to care for the dying and seriously ill with minimal resources. The resilience required for such work cannot be underestimated. While one volunteer explained, *"Through helping people I get what I want, so God blesses me with other things.... The confidence the patients have in me gives me courage"*. (Ug Vol) Others talked of the emotional cost, *"I realise that palliative care is a good service, but it is so stressful. Most of our patients come to us with advanced disease. After a few months, we will lose that patient, and it is very hard. It's painful. It helps that we are able to help them die in peace *". (Ug Vol)

### Kenya

The Maua Hospital palliative care team is staffed by four nurses and a doctor from the Hospital Community Health Department, and supported by local volunteers across a wide rural catchment area where health services are largely absent. Most patients have AIDS or cancer. Patients were often too weak to seek help, while others who did make the long journey to a hospital were sent back home with little or no treatment or support. One Kenyan volunteer after reflecting on the past, said, *"Nowadays we try to make sure that no-one dies alone and neglected in the village"*. At least now with the team presence many more people get good pain relief than previously was the case.

**Figure 2:**
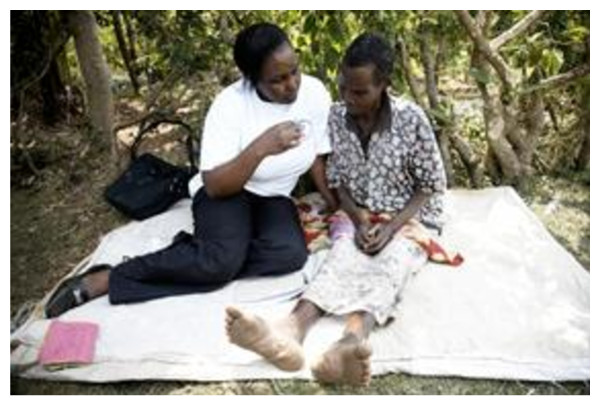
**Kuraitha (comforting and supporting*)***.

What mattered to patients was the package of care that the team delivered. The daughter of an elderly women dying of cancer said, "I *really appreciate the Maua team, even the love they showed to my mother, talking, praying, comforting her. They helped the whole family. What they did was not just gucera (visiting casually); it was kuraitha (comforting and supporting)." (Ken Carer of mother Cancer)*.  Figure 2 captures a Maua team member offering comfort and support.

Alongside providing holistic care the palliative care team enabled relatives and friends to care, taking their fear of dying away and giving them the skill they needed. One widow explained that the nurse had told her, *"It will be okay, because we are together, even if your husband is going to die*". (Ken carer of husband, HIV and cancer) An elderly widow told how she was able to look after her husband until his death, due to the team's support. *"As my husband's mouth cancer got worse, he and I went to a seminar at Maua Hospital, where I learned how to measure his medicine and give it to him. In fact, I almost became a doctor! My husband was never admitted to the hospital. He died at home, not in pain." (Ken Carer of husband, cancer)*

**Figure 3:**
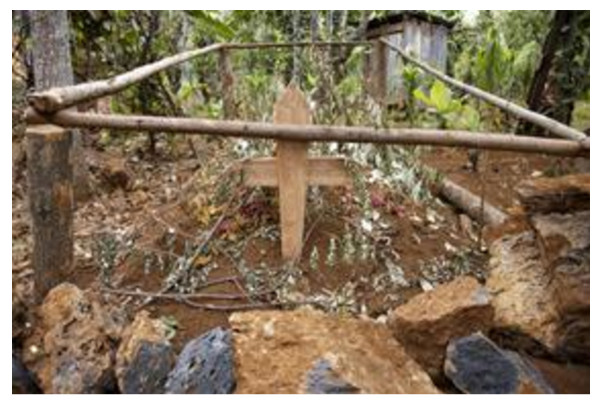
**A fresh grave in the back garden after a home death**.

Several carers spoke of the great satisfaction they felt in having cared for and buried their loved one at home in their family land, with support from the palliative team. One carer explained the practical (traditional) advice she had received about preparing the body for burial - how to preserve the body in the home by placing sacks on the floor, covering them with charcoal, smashing the charcoal, and soaking it with water. After placing the body on the charcoal, she kept the charcoal wet, so that the evaporating water would keep the body cool. Figure 3 shows the grave of a patient in the family’s garden.

One of the biggest barriers to offering good care, and to enabling patients to be cared for at home was distance from services, and lack of skilled local nurses. Mobile phones were central to empowering families to feel secure in their role as carers, with carers ringing volunteers and nurses to discuss care. They also provided volunteers with access to support to make decisions.

As with both other programmes the presence of the palliative care teams in the community had shifted attitudes towards those who are dying, enabling families and the community to provide more comprehensive care. When the Kenyan team started work people shunned talk of dying, not understanding palliative care. Many expressed the view that seeking for health care was about finding a cure, and when this was not possible, there was nothing that could be done, but now as the team leader explained: *"Even the men are now finally coming around*." (Ken, Nurse) Much of this community acceptance was due to the work with local churches and village chiefs and with local churches where pastors included time during worship to talk about palliative care.

Many community members were now knowledgeable about HIV/AIDS, but knowledge of cancer remained very low. *"People still think cancer means death that very day," *one volunteer explained (Ken Vol) Sometimes relatives refused to let the palliative team disclose the diagnosis of cancer to patients, especially very elderly patients.

A rural Kenyan pastor said, *"Some people fear to say the word 'cancer'. There's no local word for it, but even the old people and small children know that 'cancer' kills. They think the patient will lose hope and die tomorrow. There's a lot of ignorance about it. Patients go from one place to another, to charismatic sects, or to healers who take their money and give them useless things, but there's no improvement." (Ken community leader)*

The Maua programme, however, seemed to be facing special difficulties in retaining many of their volunteers. As a number of the volunteers explained, they too are just ordinary citizens, often living in poverty; they experience the same needs and demands on their time as the families they are trying to help.

Like the other programmes volunteers provided a central role in facilitating care. The commitment shown by the active volunteers was extraordinary, as one volunteer told us, *"My husband died of AIDS and I myself am infected. But I am taking anti-retroviral medicines and I have been given a chance to live and raise my children. I want to help others do the same*". ( Ken vol)

## Discussion

### Summary

All three palliative care programmes brought essential pain relief to patients, reduced physical, emotional and spiritual suffering, and supported family members to care for their patients in their home environments. Oral morphine was a vital and often new intervention which greatly benefited patients, and helped staff to deliver and know they were providing effective care. Though constructed differently, all programmes focused on delivering care which was culturally and contextually appropriate to the local conditions, and which attempted to deliver much more than pain relief on a limited budget. The programmes faced several key issues: how to provide effective palliative care in the face of poverty; how to encourage early identification of patients who would benefit; and how to develop effective and integrated referral systems. All programmes faced a new challenge of how to manage patients who were previously dying but are now surviving (due to antiretroviral therapy or successful amputations). Programmes are just beginning to recognise the vast and often hidden burden of non communicable diseases such as cancers. Programme innovation was marked by the marriage of new technologies such as mobile phones with ancient traditions such as the use of charcoal and water to preserve the dead body. National differences in how health systems were established and managed affected local differences in programme delivery. This was most evident in the different ways that HIV treatment, patient management and palliative care were delivered, with Malawi's very effective HIV programme functioning as a vertical programme.

### Strengths of study

At each site the Rapid Evaluation Methodology (REM) allowed data collection from different sources, and provided a structure for prompt data integration and analysis. Interviews with patients and carers using independent translators gave interviewees the chance to criticise the local palliative care team members. We tried to minimise a possible bias caused by programme staff selecting the patients for interviews by searching, when possible, for alternative or disconfirming cases. We did not electronically record interviews, so data recording and analysis were not exhaustive. However in each site the team completed, analysed and discussed their notes within a few hours after each observation or interview. Having a photographer on the research team sensitised all members to the physical settings and details of the homes and communities, and as data in their own right, photographs tell stories, invite interpretation, and like written quotes can be powerful and evocative [15]. In keeping with WHO rapid appraisal methods, we rigorously collected and analysed what we considered enough data to come to reliable findings and conclusions, without making it an exhaustive academic exercise [16,17].

### Weaknesses of the study

This study, in countries where a total of over one million people require palliative care, was restricted for practical reasons to only three site visits of one week each. Returning to each site to perform serial interviews would have provided longitudinal and richer information about the experience of dying and the range of interventions at different times and stages of illness. Furthermore, palliative care team members accompanied us on most visits and while they did not remain in the room during interview, their presence in the vicinity may have influenced patient, carer and community responses. Finally, we recognise that in such poverty, patients and carers were often grateful and accepting of any care at all, and therefore they may give a biased report of the importance of the palliative care team. Our data collection method focused on describing impact on individual patients and their carers in the local community context: we did not assess wider issues.

### Palliative care amid poverty

In the face of poverty, compassionate workers could not just dispense analgesia. Patients and families also needed basic food, a safe and dry place to live, blankets and sheets, and frequently school fees to prevent children being sent away from school. All three programmes were modestly funded to cover staff salaries, analgesia, travel, and volunteer training. All three advocated locally for resources to provide food and finances for people dying in poverty. Staff members, however, were often greatly distressed by their inability to provide much practical help, and they gave of themselves by taking time to listen to patients and families. It was often the volunteers, with few personal resources, who would help patients and their families buy essential food. These acts demonstrated that their concern for individuals went far beyond providing effective analgesia. Patients spoke positively of being cared for as people rather than as diseases, particularly by volunteers.

### Embedding palliative care within the community and within the health services

All three programmes struggled to embed the principles of palliative care within the community and with how to shift unhelpful attitudes to people who were dying. Patients frequently presented very late with advanced illnesses. Thus providing information about the nature of illnesses, and helping communities understand that palliative care can make a difference remain priorities, as does developing effective and timely referral systems between hospitals, clinics and palliative care teams. Given the community dependence on traditional healers, those healers should also be trained about when and how to refer patients into palliative systems.

### Embedding palliative care within the health service

Palliative care needs to be cost effective and integrated into mainstream care. At present, care systems sit in "silos" without easy access paths to one another. Patients bear the consequences of this lack of integration. Seeking curative services (either simultaneously or consecutively) from traditional healers, herbalists and health facilities drained patients and their families of almost all their resources. Current health services, were not designed to offer integrated long-term chronic care, and required patients (especially those with HIV/AIDS patients in Malawi), to shuffle among two or even three different service providers - one offering pain control, another delivering antiretroviral therapy, and still another dealing with the side effects of treatment. While the service in Uganda, demonstrated a more integrated approach with palliative care being offered as part of an overall HIV programme, integration problems remained with other illnesses. More explicit frameworks are urgently needed for service development and palliative care integration throughout the disease course [18].

### A primary health care approach using volunteers

By adopting a primary health care approach to delivery, these programmes attempted to maximise their capacity. All programmes were reliant on volunteers for case finding, ongoing social support, and end-of-life care. Volunteers, delivered much of the day-to-day care and built relationships between patients, the palliative care team, and the community. However, volunteer training and ongoing motivation are challenges in all sites (but especially so in Kenya) and are accentuated when paid staff and volunteers work alongside. The integration of palliative knowledge and understanding in the general training of village health care workers is strategic, as is the need to develop a greater support network for all volunteers.

### Palliative care must be much more than terminal care

Our findings highlight that palliative care has followed a particular path in Africa. While initial palliative care services were developed specifically for cancer such as Island Hospice founded in 1979 in Zimbabwe and Nairobi Hospice in Kenya in 1990, the HIV pandemic pushed forward a new wave of palliative care in the 1990s which was very much focussed on home based care. The more recent constructions of palliative care, particularly as defined by PEPFAR, became very much associated with terminal AIDS care. While palliative care will always be needed for those dying with AIDS, with the availability of antiretroviral therapy, the needs of some patients with HIV are changing. In Malawi, for example, some patients who were seriously ill with AIDS and were being visited regularly initially were now no longer housebound. There is a danger that the structure of programmes will lag behind the transition of HIV from a terminal to a chronic disease, or conversely that donors may see less need for palliative care. The situation is complex. Some patients with HIV currently need home-based care for the last days of life. Others require long-term comprehensive social care and rehabilitation now, but may need palliative care in the future. Neither health providers nor donors should expect that the need for palliative care in Africa will diminish. Therefore alongside the urgent need to understand the burden of dying for patients with many different illnesses, there is also an urgent need for more research to fully understand the needs of patients with HIV, especially within the changing nature of the disease so that services can be planned appropriately. Lessons from these shifting needs can be used to help reconstruct palliative care to develop a more dynamic and comprehensive approach from diagnosis of a life-threatening illness. Palliative care as part of a continuum of care, and not just for patients in the terminal stage of their disease, needs advocacy in Africa, as much as it does in developed countries [19].

### In contrast to richer countries, cancer patients receive proportionately less palliative care

Palliative care programmes in Africa need to prioritise cancer case finding. Cancer is still "hidden in the shadow of AIDS" in Africa, causing much suffering [20,21]. Curative and disease-modifying treatment is limited, and often available only to a small rich minority. In Uganda and Kenya, the palliative programmes are increasingly dealing with cancers, but are just starting to scratch the surface. The Kenyan site studied, for example, serving a catchment area of 700,000 people, only had 50 cancer patients currently being reached by the palliative care programme. This is less than 10% of the estimated number with advanced cancer. Cancer is stigmatised, a disease that carries a strong message of death, yet there is little understanding of this stigmatisation and how best to approach it. Further research is needed to interpret how such messages can be challenged.

### Resourceful approaches to technology: the case of mobile phones

**Figure 4:**
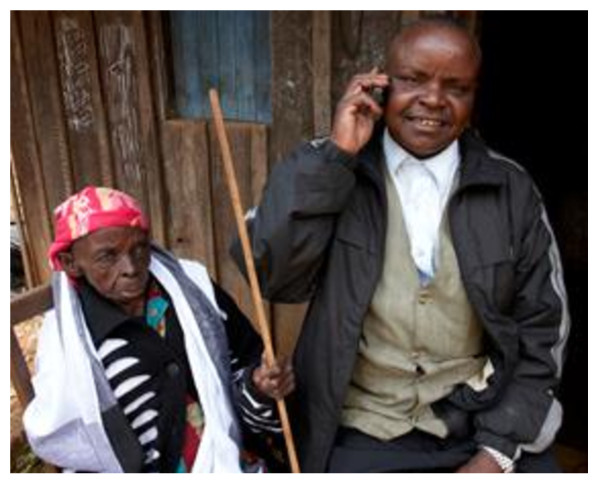
**A son ensuring continuity of quality care through a mobile phone**.

We were struck by the fact that many health workers, even in remote areas, are at the cutting edge of adapting new technologies to meet local needs. Mobile phones, almost unknown in these countries 15 years ago, are now used by families, volunteers and programme staff to provide a sophisticated form of tele-health. They are relatively inexpensive, but airtime is costly, so the projects encourage families and volunteers to "flash" a nurse on their cellphones as illustrated in Figure 4. Thus volunteers were mentored, and access to competent care was greatly expanded. Even in homes with no running water or electricity, mobile phones permitted health communication and support.

Side by side with this use of technology, programme staff also encouraged families to use old and traditional methods of care. For example, the use of charcoal for preserving a body enabled families to care for their members at home until death and for a few days afterwards, thus eliminating the need to die in a hospital, where an expensive morgue could hold the body until relatives arrived from afar for the funeral.

### Further work

Further work is necessary to identify and test models of care which feature a number of the factors and outcome measures identified above as important for patients and carers. Such interventions could be carried out using a cluster randomised design, or staged intervention, allowing rigorous evaluation.

## Conclusion

Palliative care must address total pain and suffering across all dimensions. Widely accessible and effective analgesia is a great unmet need in Africa, but morphine in isolation is not enough. These three programmes work in the context of poverty by attempting to retain a holistic approach, acknowledging patients' great physical and social needs, and providing psychological and spiritual support. While the HIV pandemic has created a much wider global awareness of the need for palliative care, those living with other life-limiting illnesses such as cancers have less access to help.

Programmes must be configured to respond to local needs and customs, be community based, and be integrated with local health and social care, with accessible referral pathways between and across services. They also must be responsive to changing individual and population needs and to shifts in local and national health structures. While these three programmes greatly benefit a fortunate few in each area, a public health approach with integration into government policy and core health service provision is necessary to significantly impact the national burden of palliative care needs in each country.

### Consent

Signed consent for every photograph was requested and received by the professional photographer before any photographs were taken. Patients choice not to be photographed was respected. All signed consent papers are lodged with the Diana Princess of Wales, Palliative Care Initiative.

### Ethical approval

This project was a "service evaluation" rather than experimental research and as such did not require formal ethical approval. http://www.nm.stir.ac.uk/documents/SouthEastScotlandResearchEthicsService.pdf

## Competing interests

The authors declare that they have no competing interests.

## Authors' contributions

LG, SAM, ML, JB designed the study. All authors were involved in data gathering and analysis, NB using photography. All the authors have participated in data interpretation and writing the manuscript. SM is the guarantor.

## Pre-publication history

The pre-publication history for this paper can be accessed here:

http://www.biomedcentral.com/1472-684X/10/8/prepub
